# Predicting Cutting Force and Primary Shear Behavior in Micro-Textured Tools Assisted Machining of AISI 630: Numerical Modeling and Taguchi Analysis

**DOI:** 10.3390/mi13010091

**Published:** 2022-01-07

**Authors:** Shafahat Ali, Said Abdallah, Salman Pervaiz

**Affiliations:** Department of Mechanical and Industrial Engineering, Rochester Institute of Technology—Dubai Campus, Dubai P.O. Box 341055, United Arab Emirates; sa2654@rit.edu (S.A.); sma6163@rit.edu (S.A.)

**Keywords:** machining, finite element analysis, FEA, microtextured tools, AISI 630

## Abstract

The cutting tool heats up during the cutting of high-performance super alloys and it negatively affects the life of the cutting tool. Improved tool life can enhance both the machinability and sustainability of the cutting process. To improve the tool life preferably cutting fluids are utilized. However, the majority of cutting fluids are non-biodegradable in nature and pose harmful threats to the environment. It has been established in the metal cutting literature that introducing microgrooves at the cutting tool rake face can significantly reduce the coefficient of friction (COF). Reduction in the COF promotes anti-adhesive behavior that improves the tool life. The current study numerically investigates the orthogonal cutting process of AISI 630 Stainless Steel using different micro grooved cutting tools. Results of the numerical simulations point to the positive influence of micro grooves on tool life. The results of the main effects found that the cutting temperature was decreased by approximately 10% and 7% with rectangular and triangular micro grooved tools, respectively. Over machining performance indicated that rectangular micro groove tools provided comparatively better performance.

## 1. Introduction

Several researchers have focused their work to explore the machining performance of microtextured tools. The machining of high-performance alloys is quite challenging in nature, and due to this it provides poor surface finish and tool wear. Zheng et al. [1] investigated the performance of micro-textured tooling for the machining of Ti6Al4V. The effect of the cutting force, cutting speed and depth of cut at different micro-textured tools was observed. In the study, the authors observed four different micro-textured cutting tools. The study observed that the sinusoidal micro-texture tool gives best performance over these parameters. By using this texture, they observed that the surface roughness decreased by 35.89%. Mishra et al. [2] conducted simulation and the experimental work on the Ti-6Al-6V with cutting tools using texture and texture with coating of PVD-TiAlN. Authors used the AdvantEdge software to perform the 2D simulation to observe the performance. The research used the novel chevron texture, and studied the effectiveness of using this tool on the cutting forces, chip contact length and shear stress. The better performance observed by the coated texture tool showed a more than 30% reduction in the chip contact length and around 17% reduction in the forces.

Several lubricants harm the environment and react with the metal to form oxides that are not good for the work piece. It is necessary to introduce the concept of sustainability in the field of machining. Krolczyk et al. [3] used the technique to minimize the lubricant to avoid harmful effects on the atmosphere. The study used vegetable oil or nanofluid lubricants to overcome the adverse effect of the lubricants. They used the minimum quantity lubrication (MQL) technique to avoid the extensive used of lubricants and to sustain our ecosystem. The study was performed for the integration of the cutting tool and coolant to achieve the optimal result. The study used a laser-based cutting tool with normal vegetable oil cutting fluid and nano particle fluids under an nMQL environment. Performance was then compared with the dry cutting. The study observed the reduction in the tool chip contact length, friction, and adhesion of tool material with the work material when textured tools were employed. Chen et al. [4] studied the material behavior of the Ti-6Al-6V using different conditions under the orthogonal machining. The study investigated chip formation and energy density using finite element modeling. The study discussed the plastic cutting model and influence of the segmented chip formation at different cutting speeds.

Lu et al. [5] provided a two-dimensional finite element model for the orthogonal cutting. Two different criteria were used to understand the chip formation and plastic behavior in machining. Simulations were performed by changing different parameters, like cutting speed, rack angle and depth of cut. The study reported two types of fracture that occurred at the edge of the surface (opening mode crack) and sliding mode crack. The study recommended optimized angles and speed for the cutting process. Mishra et al. [6] studied the different texture with different area density and depth. They used four different geometrical shapes—circular, square, triangular, and elliptical—under the dry cutting condition. The study found that, when using a micro-textured tool and chip, the contact length decreases but this is not applicable at the higher feeds. The most promising feature is the texture area density in the finite element (FE) simulation. Alagan et al. [7] investigated the micro-texture coupled with the high-pressure coolant. They put the texture on the rack and flank face, and it was observed in experimentation that the cylindrical dimple on the rack and square pyramidal on the flank face was more effective. The study showed that the tool life increased by around 30% when using this technique. Some material was found to be adhesive at the side, but it did not affect the cutting process.

Machai et al. [8] studied grooving operation. The study sprayed the snow CO_2_ as a coolant to the work material at the tool tip through internal channel. The experimentation found that the tool wear decreased, and the tool life increased up to two times. The result was found to be valid at a higher cutting speed as well for the notch wear or flank wear of the cutting tool. Rathod et al. [9] used a coated tool with Tungsten Disulphide coating and made the micro-texture on the rake face. The study used square and circular texture tools, and also study observed that tool material adhesion was approximately 60% reduced for the square texture. The cutting force decreased using textured tools. Lower friction also reduced the cutting temperature and plastic deformation of the cutting tool’s work material. Zhang et al. [10] studied the combined effect of micro-textured tools with magnetic nano particles under the magnetic field. Micro-textured tools were made by the laser texturing technique and nano particles were prepared with 30% solid content. By applying these conditions experimentally, the study found around a 50% reduction in the cutting force and the same for the surface roughness of the material. The study compared the results using the same experimental procedure for the simple cutting tool and coolant. Hoa et al. [11] discussed the machining of hard material. Their research work employed a hybrid technique using variable density texture, variable shape texture, variable shape and density texture. The study used textured and non-textured tools for the machining of the titanium alloy in the dry cutting and minimum lubricant. After performing experiments, the study found that the variable shape and density texture provided the most promising features in the result of tool wear, friction forces and reduced trust force.

Lian et al. [12] studied the ball on the disk for different texture and found the optimal textured tools using the FEA simulations. The adhesion of the work material was a serious defect using simulations. The study used a solid lubricant and specific diameter for texture, depth and spacing under orthogonal cutting. It was found that tool wear and surface adhesion was decreased by increasing the space between the texture. Duan et al. [13] studied the derivative cutting of the material. It occurred when machining is performed at the bottom side of the chip, and it hindered the effectiveness of the textured the tool under the dry cutting condition. The derivative cutting increased the pressure on the rack side of the cutting tool which increased force and tool wear. Ranjan et al. [14] provided the review of different machining process using textured and non-textured tools. The study provided a detailed review of different studies about different set of parameters used in machining. The study pointed out that nano-textured tools produced better results than other types of tools. Kawasegi et al. [15] studied optimization of the shape of the diamond cutting tool by having texture on it. The texture effect was studied by changing the different machining parameters, such as cutting speed and feed. The study found that chip contact length was affected by the shape of texture.

In this study, laser textured cutting tools were tested for the machining of 6061 aluminum tubes. Xing et al. [16] investigated the cutting forces, friction coefficient, chip compression ratio, and surface quality to determine output performance parameters. Researchers found that rectangular grooves produced the most effective results compared to liners, circular grooves, and conventional tools. Xing et al. [17] investigated the performance of microtextured cutting tools with three different geometries (linear grooves parallel to cutting edge, wavy grooves, and linear grooves perpendicular to cutting edge). In addition, nanoscale textures are applied to the tools and the results are compared with conventional tools for the machining of hardened steel. To determine the performance of the textured tool, different performance parameters, such as cutting temperature, cutting forces, tool chip interface, and friction coefficient, were measured. As compared to other textured and conventional tools, the wavy-textured tools showed the greatest reduction in the performance parameters.

The textured tool was tested in situ at different locations along the rectangular groove to investigate its performance. Sugihara et al. [18] measured primary and secondary deformation parameters with grooves at different locations and with a normal cutting tool. The cutting tool was grooved at three different locations. The grooves are located in region 1, where the sliding friction is dominant, in region 2, where the sticking friction is dominant, and in region 3, which is close to the cutting edge. Region 2 shows a 60% decrease in friction force, followed by region 1 with a 30% decrease. There was no advantage to region 3 over the normal and other regions of groove tools. Ma et al. [19] developed a finite element simulation to compare the performance of the micro bump textured tool with the normal or non-textured tools when machining AISI 1045 steel. The effect of micro bump parameters, such as height, width, and distance from the edge, are studied in order to improve machining performance. Three parameters are analyzed: the main force, the thrust force, and the chip tool contact length. Using these parameters, they found that the main force and trust force had decreased by more than 10% and 20%, respectively.

The current study investigated the performance of micro-textured tools using triangular and rectangular textures for the machining of AISI 630 stainless steel. 

## 2. Numerical Design

### 2.1. Simulation Setup and Taguchi Design of Experiment (DoE)

**Figure 1:**
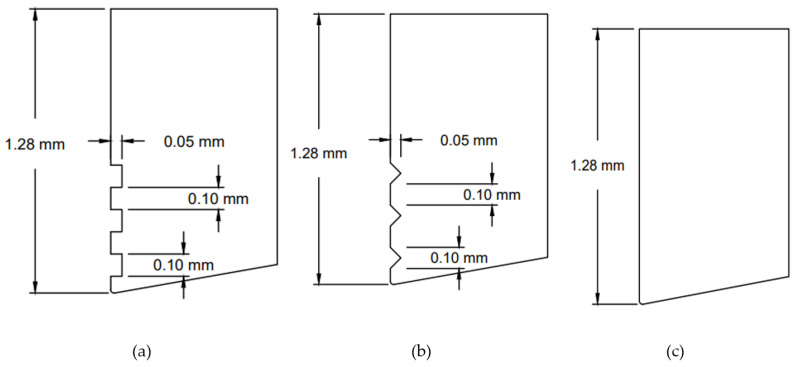
Microgrooves on the cutting tool, (**a**) rectangular (**b**) V-shaped or triangular and (**c**) flat tool.

For finite element model (FEM) simulation the work material is stainless steel and using the carbide tool material. The parameters of cutting speed, feed, and microgrooves were selected in this simulation work. The simulation was performed under the flooded environment in the AdvantEdge software. The parameters varied from 75,150 and 225 m/min for cutting speed, 0.1, 0.2 and 0.3 mm/rev for feed rate. Three types of micro-groove tools, such as flat, rectangular, and triangular, were used. The Taguchi design with orthogonal array of L9 against 3 × 3 experiments were used in this work. Figure 1 shows the schematic illustration of the micro-grooved tools. Parameters for the microgrooves are given below in the Table 1. The Taguchi design followed for the simulation is provided in Table 2.

### 2.2. Material Model

Material modeling of AISI 630 was performed using the advanced power law-based formulation available in the AdvantEdge software. The advanced power law consists of three terms to mimic the behavior of strain hardening thermal softening and rate sensitivity. Strain hardening is represented by the function of *g* ($\varepsilon^{p}$). Thermal softening is represented by the function of *θ*(*T*). Strain rate sensitivity was included by the function *τ*($\dot{\varepsilon }$). Equation (1) shows the advanced power law [19,20,21,22].

(1) $$\sigma \;(\varepsilon^{p},\;T,\;\dot{\varepsilon })\;=\;g\;(\varepsilon^{p})\;\theta (T)\;\tau (\dot{\varepsilon })$$

Equation (2) is used to develop the function of the strain hardening function. Experimental data is curved fitted to get the parameters of Equation (2). Parameters of Equation (2) were selected from the available literature [21]. In Equation (2), *σo* shows the initial yield stress, $\varepsilon^{p}$ represents plastic strain, $\varepsilon_{o}^{p}$ shows reference plastic strain, and 1/*n* is described as strain hardening power. 

The strain hardening is included in the material model using Equation (2). The parameters of Equation (2) were identified by the stress strain curves of the material and curve fitting. Equations (3) and (4) incorporate strain rate sensitivity and thermal softening [19,20,21,22].

(2) $$g(\varepsilon^{p})=\sigma o\;{\left[1+\frac{\varepsilon^{p}}{\varepsilon_{o}^{p}}\right]}^{\frac{1}{n}}\;\;\;if\;\varepsilon^{p}\;<\;\varepsilon_{cut}^{p}$$

where *σo* displays initial yield stress, $\varepsilon^{p}$ indicates plastic strain, $\varepsilon_{o}^{p}$ demonstrates reference plastic strain, and 1/*n* is designated as strain hardening power.

(3) $$\tau (\dot{\varepsilon })=\sigma o\;{\left[1+\frac{\dot{\varepsilon }}{\dot{\varepsilon }o}\right]}^{\frac{1}{m1}}$$

where $\dot{\varepsilon }$ depicts plastic strain rate, and ${\dot{\varepsilon }}_{o}$ describes reference plastic strain rate and *m*_1_ shows strain rate sensitivity.

*θ*(*T*) = *c*_0_ + *c*_1_*T*^1^ + *c*_2_*T*^2^ + *c*_3_*T*^3^ + *c*_4_*T*^4^ + *c*_5_*T*^5^
*if T* < *T_cut_* (4)

(5) $$\theta (T)\;=\;\theta (T_{cut})\;(1-\frac{T-T_{cut}}{T_{m}-T_{cut}})\;if\;T\;>\;T_{cut}$$

Thermal softening function is depicted in Equations (4) and (5). In AdvantEdge, the thermal softening function is defined by a polynomial function with C1–C5 constants. These constants C1–C5 were obtained by a curve fitted by the compression test data at higher temperatures. Where *T* is the temperature during the test, *T_m_* is the melting temperature, and *T_cut_* is the cut-off temperature.

**Figure 2:**
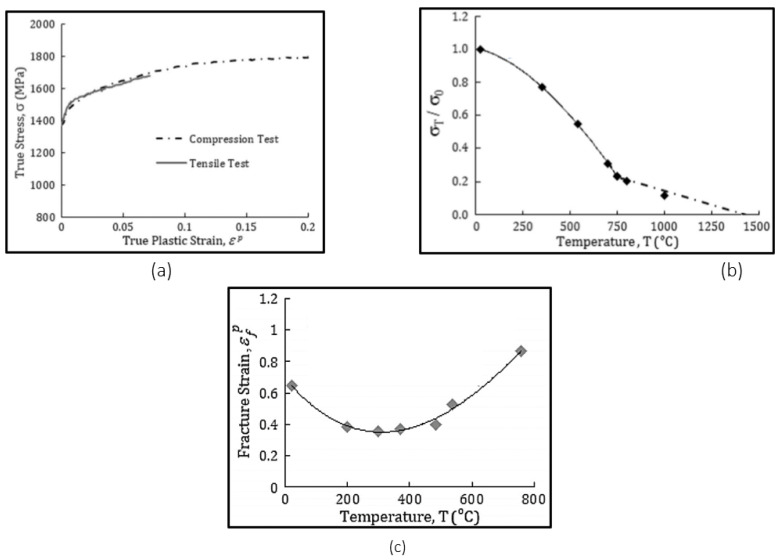
For material AISI 630 stainless steel, (**a**) true stress strain curve, (**b**) thermal softening curve, (**c**) fracture strain curve (adopted from [21]).

Figure 2 has the benchmarked data from the literature that was used to obtain the material coefficients. Friction at the tool chip interface is addressed by the sliding friction model, where the sliding force and normal load is directly proportional to each other, as represented under in Equation (6). The constant of proportionality is referred to as the coefficient of friction (*µ*). As per the literature, the value of the coefficient of friction was taken as 0.5 for all simulations.

*τ* = *μ*
*σ_n_* (6)

Chip formation is produced by the damage function (*D*) as shown in Equation (7). The fracture strain can be represented using the temperature-dependent model, as shown in Equation (8) below [19,21].

(7) $$D=\sum_{i}\frac{\Delta \varepsilon_{i}^{p}}{\varepsilon_{f_{i}}^{p}}$$

(8) $$\varepsilon_{fo}^{p}=d_{0}+d_{1}T^{1}+d_{2}T^{2}+d_{3}T^{3}+d_{4}T^{4}+d_{5}T^{5}$$

The AdvantEdge software manual and literature [19,21] were consulted to obtain all of the required parameters for the AISI material model.

## 3. Results and Discussion

### 3.1. Cutting Temperature

**Figure 3:**
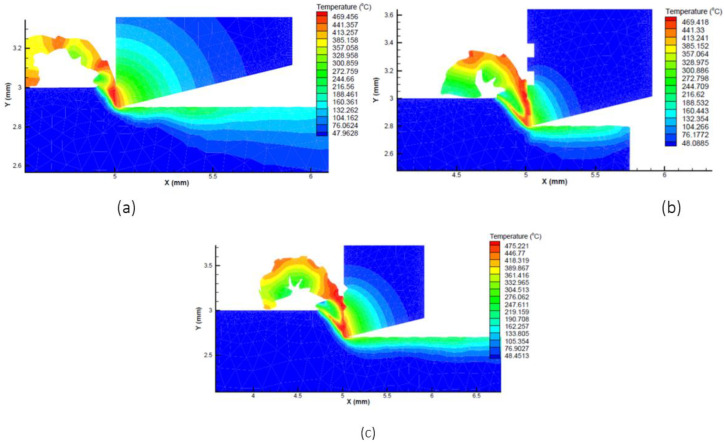
Simulation of flat, rectangular, and triangular microgroove tools at cutting speed of 75 m/min and feed of (**a**) 0.1 mm/rev (**b**) 0.2 mm/rev and (**c**) 0.3 mm/rev.

**Figure 4:**
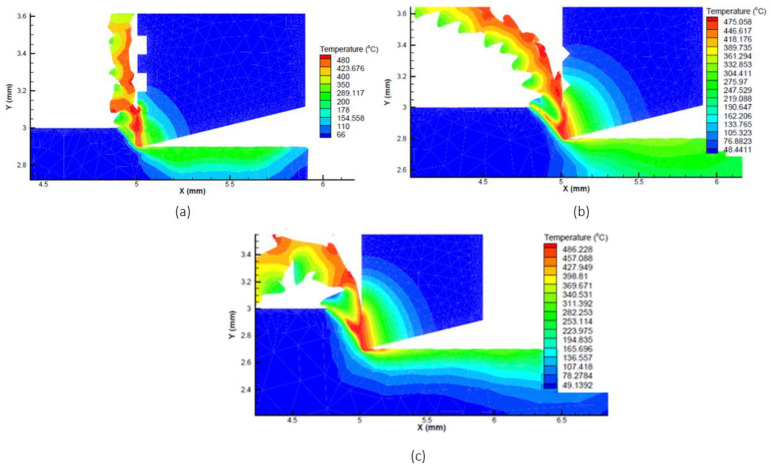
Simulation of flat, rectangular, and triangular microgroove tools at cutting speed of 150 m/min and feed of (**a**) 0.1 mm/rev (**b**) 0.2 mm/rev and (**c**) 0.3 mm/rev.

The simulations were performed on the AdvantEdge software and the result of the cutting temperatures were evaluated. From the figure, the highest temperature found during the cutting operation occurred on the tip of the cutting tool, which varies according to different microgrooves, cutting speed and feed rate. Cutting temperature responses were gathered from the simulated data and were analyzed further using the signal to noise ratio, mean plots and analysis of variance (ANOVA). The resulting simulations are represented in Figure 3 and Figure 4 against cutting speeds of 75 m/min and 150 m/min. These figures also showed how the cutting temperature varies with changing conditions. 

**Figure 5:**
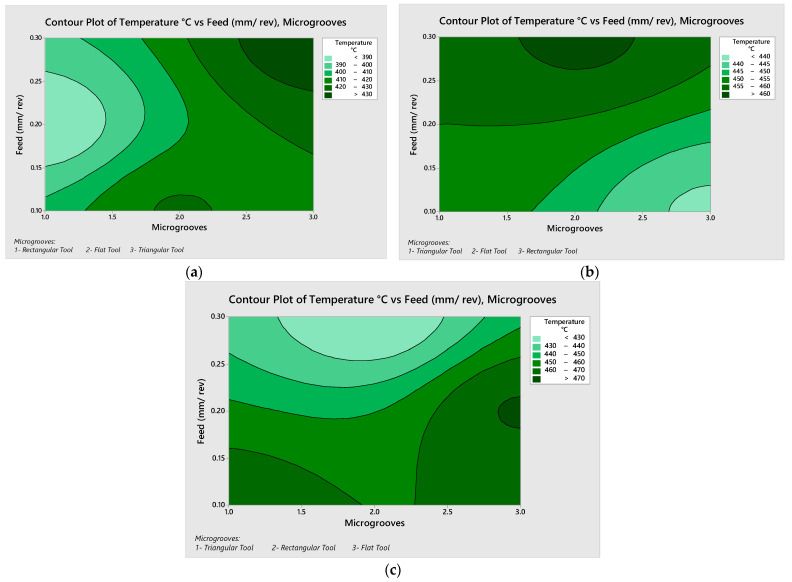
Contour plots for the cutting temperature (**a**) 75 m/min (**b**) 150 m/min and (**c**) 225 m/min.

The simulated data contour plots of the cutting temperature with respect to the different micro-grooves were also plotted, as shown in Figure 5. Contour plots are very useful to show the relationship between three parameters, where input variables are plotted on the x and y scales and the response variable is plotted on the XY plane in the form of contours. In Figure 5a, rectangular micro grooved tools provided the lowest cutting temperature of 382 °C at 75 m/min and 0.2 mm/rev. The highest cutting temperature contours were found in the lower middle part and upper right corner of the contour plot. This points at the generic trend, that increasing the feed rate increases the cutting temperature. At 75 m/min triangular micro-grooved cutting tools provided the highest comparative cutting temperatures. The cutting temperature for rectangular micro-grooved tools was found to be 10% lower, comparatively. Similarly Figure 5b,c showed respectively lower values of cutting temperature for the rectangular micro-grooved tools. From the above discussion, it is concluded that the use of rectangular microgrooves decreases the temperature of the cutting tool by almost 10%, as compared with the flat cutting tool at higher feed or depth of cut excluding the 150 m/min condition where it showed a decrease of 4%.

Furthermore, for the cutting speed of 225 m/min, the cutting temperature was 470.71 °C and 421.55 °C for feed rates of 0.3 mm/rev and 0.3 mm/rev, respectively, for the flat tool. For the cutting speed of 150 m/min and 0.2 mm/rev feed rate the triangular grooved tool showed the temperature of 455 °C and the normal tool showed 461.25 °C. In summary, this triangular groove reduces the temperature by around 1.5% on average. Another generic trend was observed that increasing the cutting speed results in increasing the cutting temperature, which is found following the metal cutting temperature. As the feed rate and cutting speed increase, frictional resistance increases, causing the temperature to rise.

**Figure 6:**
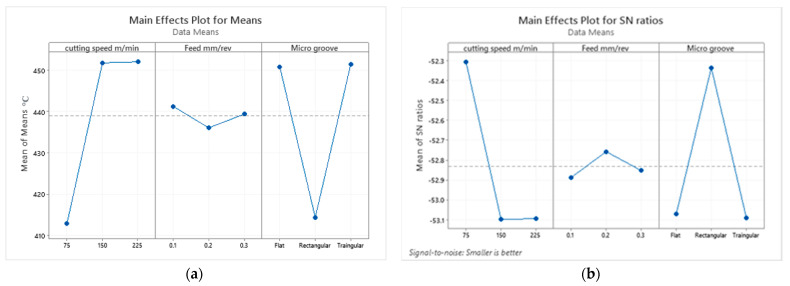
(**a**) Mean and (**b**) signal to noise plots for temperature values.

Table 3 and Table 4 and Figure 6 provide signal to noise ratio and mean response plots for the cutting temperature as output responses against the feed rate, cutting speed and micro grooved tools. It can be observed that the best performance was achieved by the cutting speed of 75 m/min, feed rate of 0.2 mm/rev and rectangular micro-grooved cutting tool. Cutting speed has the most significant influence on the output parameters, followed by microgrooves and feed rate.

The signal to noise ratio (SN) and mean response data show that the microgrooves are, on the rank, second which means that microgrooves are the most promising factor for decreasing the temperature of the cutting tool, after the cutting speed. However, the feed rates are affected the least by the cutting temperature of the tool. After Taguchi analysis, it was determined that the microgrooves affect the cutting temperature at the second rank but the exact number or percentage of that effect was unknown. For this purpose, ANOVA was executed to get the exact effects of these microgrooves on the temperature of the cutting tool. The result of ANOVA showed in the Table 5 shows that the microgrooves affect the temperature of the tool by 43.29%, while the cutting speed showed the effect of 48.72%, which is a little higher than the microgrooves.

### 3.2. Chip Ratio and Shear Angle

At the start, the chip ratio is discussed in terms of how it is affected by changing the parameters. In the experiment, the chip morphology is serrated chips, and thus the formula for the chip ratio is Equation (9) [19,20]. Where $t_{o}$ is the undeformed chip thickness or depth of cut and $t_{c}$ is the deformed chip thickness, $t_{o}$ and $t_{c}$ are measured in mm.

(9) $$r=\frac{t_{o}}{t_{c}}$$

The shear angle in the orthogonal cutting is calculated using Equation (10) [19,20]. Here, α is orthogonal rake angle and is measured in degrees.

(10) $$Tan\emptyset =\frac{Cos\alpha }{r-sin\alpha }$$

**Figure 7:**
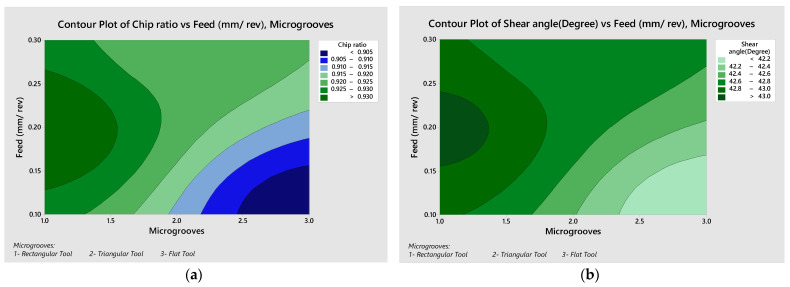

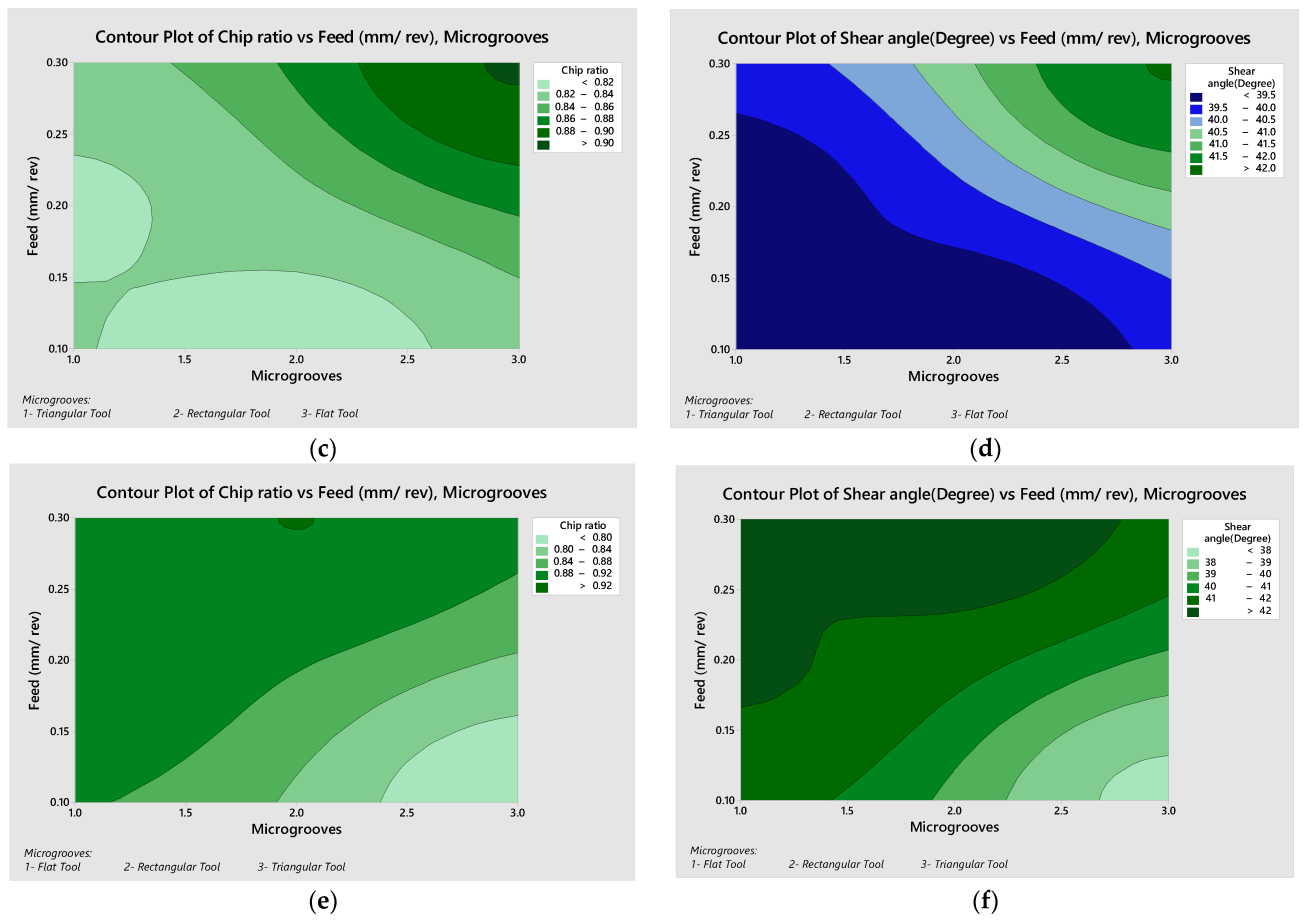
(**a**) Chip Ratio—Microgrooves vs. Feed rate at Cutting Speed of 75 m/min, (**b**) Shear Angle–Microgrooves vs. Feed rate at Cutting Speed of 75 m/min, (**c**) Chip Ratio—Microgrooves vs. Feed rate at Cutting Speed of 150 m/min, (**d**) Shear Angle—Microgrooves vs. Feed rate at Cutting Speed of 150 m/min, (**e**) Chip Ratio—Microgrooves vs. Feed rate at Cutting Speed of 225 m/min, (**f**) Shear Angle—Microgrooves vs. Feed rate at Cutting Speed of 225 m/min.

The contour plots of chip ratio and shear angle were plotted, as shown in Figure 7. At the cutting speed of 75 m/min, the highest values of the chip ratio and shear angle were located at the middle left side of the contour plots in the Figure 7a,b. This location attributes with the rectangular micro grooved tool, with medium level of 0.2 mm/rev feed rate. At the cutting speed of 150 m/min, the highest values of chip ratio and shear angle were located at the upper right side of the contour plots in Figure 7c,d. This location attributes with the flat tool with higher level of 0.3 mm/rev feed rate. At a cutting speed of 225 m/min, the lowest values of chip ratio and shear angle were located at the lower right side of the contour plots in the Figure 7e,f. This location attributes with the triangular tool with lower level of 0.3 mm/rev feed rate. Table 6 shows the calculations of chip ratio and shear angle.

Shear angle is dependent on the rack angle and the chip ratio. Here, in the orthogonal cutting rack the angle is zero and thus it is totally dependent on the chip ratio, which depends on the cutting parameters. The lower feed rates provided the lower chip ratio. Similarly, the same generic trend is seen for the shear angle. The lower value of the shear angle represents the thicker chips in the metal cutting process. The lower value of the shear angle increases the tool–chip contact length as well.

### 3.3. Cutting Force and Power Consumption

**Figure 8:**
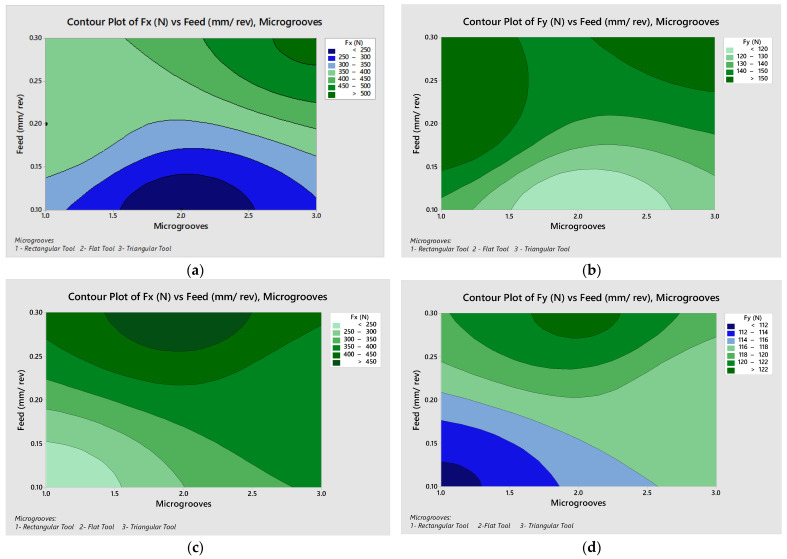

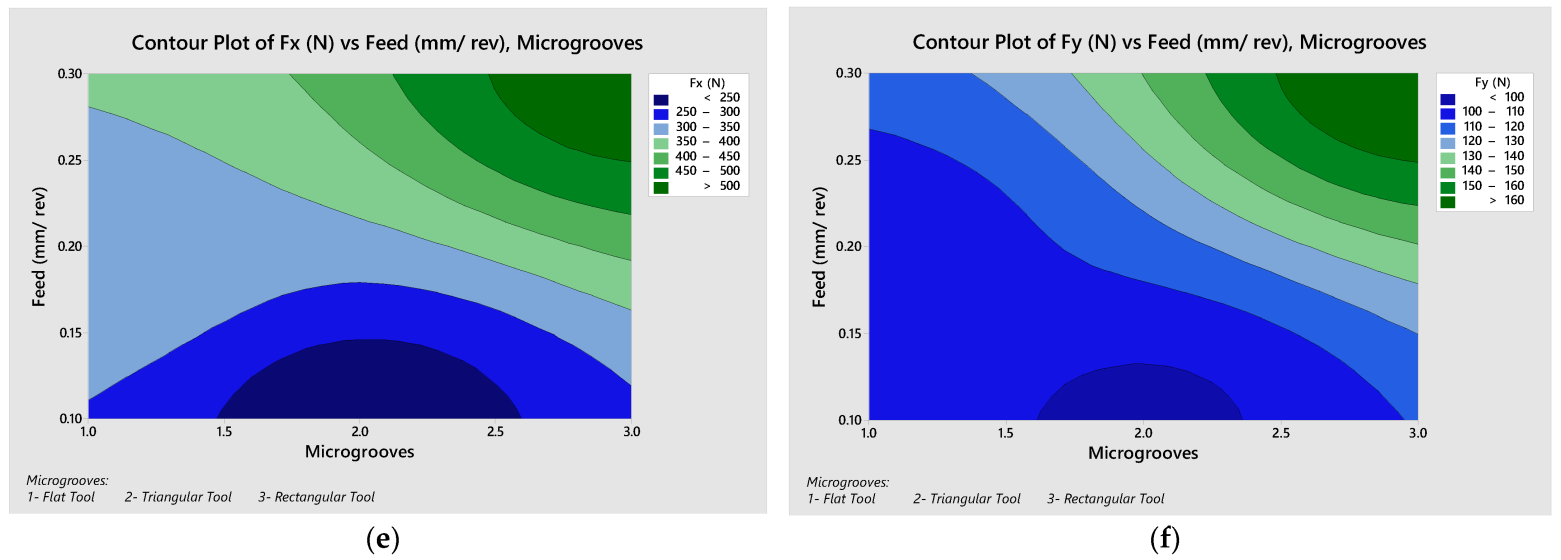
Contour plots for the cutting force components: (**a**) *Fx*—Microgrooves vs. Feed rate at Cutting Speed of 75 m/min, (**b**) *Fy*—Microgrooves vs. Feed rate at Cutting Speed of 75 m/min, (**c**) *Fx*—Microgrooves vs. Feed rate at Cutting Speed of 150 m/min, (**d**) *Fy*—Microgrooves vs. Feed rate at Cutting Speed of 150 m/min, (**e**) *Fx*—Microgrooves vs. Feed rate at Cutting Speed of 225 m/min, (**f**) *Fy*—Microgrooves vs. Feed rate at Cutting Speed of 225 m/min.

The cutting force components in x and y directions are represented at different cutting speeds and feed rates in Figure 8 below. The feed rate varied from 0.1–0.3 mm/rev, and the cutting speeds were in the range of 75–225 m/min. For the cutting speeds of 75–225 m/min, it was observed that the cutting forces increased with an increase in the feed rate and depth of cut. The main reason for the increase in the cutting speed due to the feed rate is the stain hardening phenomenon of the work material, and as a result the material becomes more difficult to machine. In the cutting process, the material is machined in plastic that facilitates strain hardening phenomenon. In addition, it was seen that the power increased with the increase in the feed rate. The same reason is valid here, that due to strain hardening, the material is more difficult to machine and more power is needed to perform the operation.

By performing the experiment on the software, the cutting power is determined from it and the other parameters are determined from the software are forces and temperature. For calculation of the shear force, we use the following Equations (11)–(13) [19,20].

(11) Fs=Fx Cos∅−Fy Sin∅

(12) $$Vs=V\frac{Cos\alpha }{Cos\left(\emptyset -\;\alpha \right)}$$

(13) Ps=Fs×Vs

where *Fs* is shear force, *Fx* is main cutting force component or tangential force, *Fy* is thrust force component, *Vs* is shear velocity, *V* is cutting speed and *Ps* is the shear power in Equations (14)–(16) [19,20]. Here, *Fs*, *Fx* and *Fy* are measured in Newton, *Vs*, *V* are measured in m/min and *Ps* are measured in Watt. 

The friction power is the calculated by multiplying the chip velocity by the friction force. The formulas for the friction force and chip velocity are given below,

(14) Fr=Fx Sinα+Fy Cosα

(15) $$Vf=V\frac{Sin\emptyset }{Cos\left(\emptyset -\;\alpha \right)}$$

(16) Pf=Fr × Vf

where Fr is the friction force, *Vf* is the chip velocity, *Pf* is the friction power, *Pc* is the cutting power, and *Pt* is the total power in Equations (14)–(16). The total cutting power is the addition of all of these values. Here, *Fr* is measured in Newton, *Vf* is calculated in m/min and *Pc*, *Pt* and *Pf* are calculated in Watt.

From the calculations in Table 7, it is clear that the cutting forces are increased when increasing the feed rate and cutting speed. The main reason here for the increase of the cutting power is due to the strain hardening of the material, which makes the material harder to cut.

**Figure 9:**
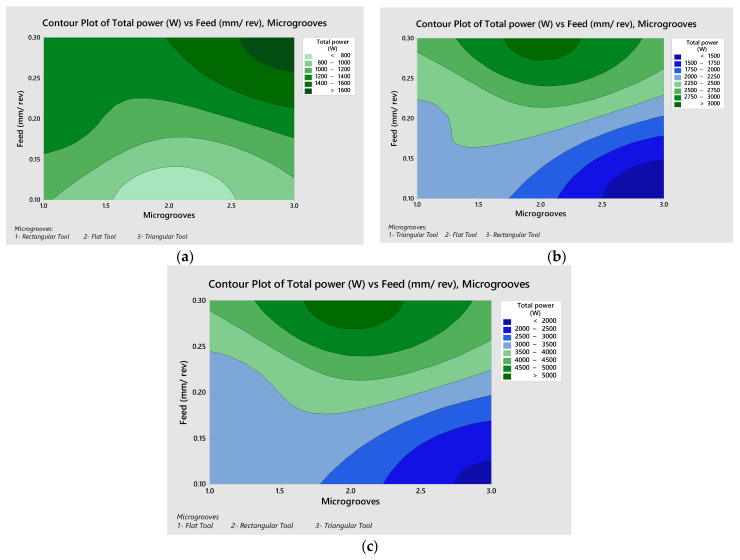
Contour plots for the total power: (**a**) 75 m/min, (**b**) 150 m/min, and (**c**) 225 m/min.

Figure 9 represents the contour plots of the total power with feed rates at different cutting velocities, ranging from 75–225 m/min. As shown in Figure 9a, at 75 m/min the higher total power was observed at the upper right corner, representing the triangular tool at a higher feed rate. The lowest total power was observed against the flat tool at a lower feed rate. In Figure 9b, at 150 m/min the rectangular tool at the lower feed rate provided optimized power. In Figure 9c, at 225 m/min the triangular tool at the lower feed rate provided optimized power. Table 8 and Table 9 shows S/N ratio and mean plots for power.

As shown in the SN and mean values, it is clear that the microgroove effecting the total cutting power was on the rank second. This means that microgrooves are a promising factor to reduce the value of the power after feed rates. As such, from all of the above data, it can be seen that the rectangular groove showed the best result over the other grooves. Additionally, the feed rate is the second most important factor for the cutting power, and the cutting speed has a minor effect.

**Figure 10:**
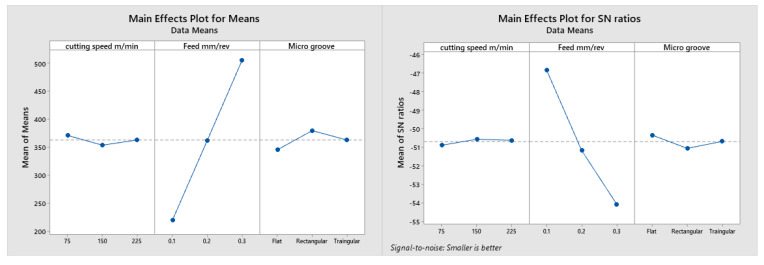
Mean and signal to noise plots for total cutting power values.

Figure 10 provides the signal to noise ratio and mean response plots for the cutting power as the output response against the feed rate, cutting speed and micro-grooved tools. It can be observed that the best performance with respect to power was achieved by the cutting speed of 150 m/min, feed rate of 0.1 mm/rev and flat cutting tool. Table 10 showed that the ANOVA technique is used to find the exact value of the percentage of the effecting factor on the total cutting power. The feed rate contributes more than 98% in power and microgroove effects, at 0.94%. Therefore, by using different microgrooves, the total power for cutting is reduced, even at higher feed rates and cutting speeds.

### 3.4. Effect of Cutting Speed vs. Microgroove on Different Parameters

**Figure 11:**
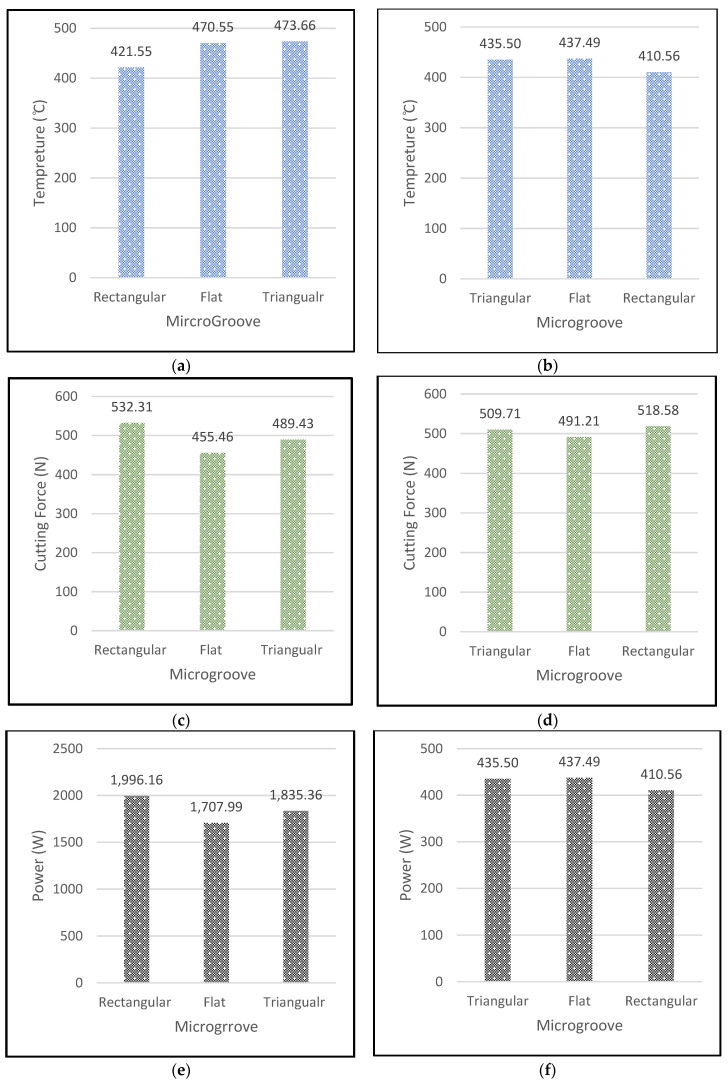
At feed rate of 0.3 mm/rev: (**a**) cutting temperature at v = 225 m/min, (**b**) cutting temperature at v = 75 m/min, (**c**) cutting force (*Fx*) at v = 225 m/min, (**d**) cutting force (*Fx*) at v = 75 m/min, (**e**) cutting power (W) at v = 225 m/min, (**f**) cutting power (W) at v = 75 m/min.

For the purpose of exploring the temperature, cutting force and power at different microgrooves, but at the same feed rate and cutting speed, these experiments were performed. As shown in Figure 11a,b, the cutting temperature at the rectangular microgroove showed the optimum result against the feed rate of 0.3 mm/rev at cutting speeds of 75–225 m/min. As per Figure 11, the cutting force and cutting power was found to be more optimized in the conventional flat tools. 

Table 11 showed the values obtained for forces, temperature and power. The temperature result for the lower feed rate of 0.1 mm/rev and cutting speed of 75 m/min in the rectangular groove showed a drop-in temperature of above 6%. Therefore, this is the process for the extraction of the heat from the tool. The forces in both the X and Y directions at both high feeds, high cutting speeds and lower feeds and lower cutting speeds showed the same result for these. The cutting forces are increased in the material due to the strain hardening phenomena which increase the amount of forces on the tool. Similarly, the power consumption is also increased in the rectangular microgrooves due to the deformation of material above the plastic region. The rectangular microgrooves even showed the same cutting temperature at a higher cutting speed, but the power decreased considerably, by around 60%, when the cutting operation was performed at the lower speed. On the other hand, the forces in the X and Y directions did not show an effective result by changing the cutting speed. Similarly, same trend was observed for other grooves at these conditions. 

### 3.5. Effect of Feed Rate vs. Microgrooves on Different Cutting Parameters

**Figure 12:**
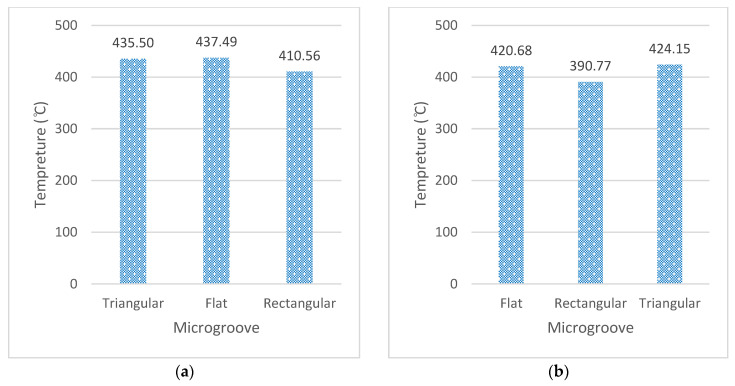

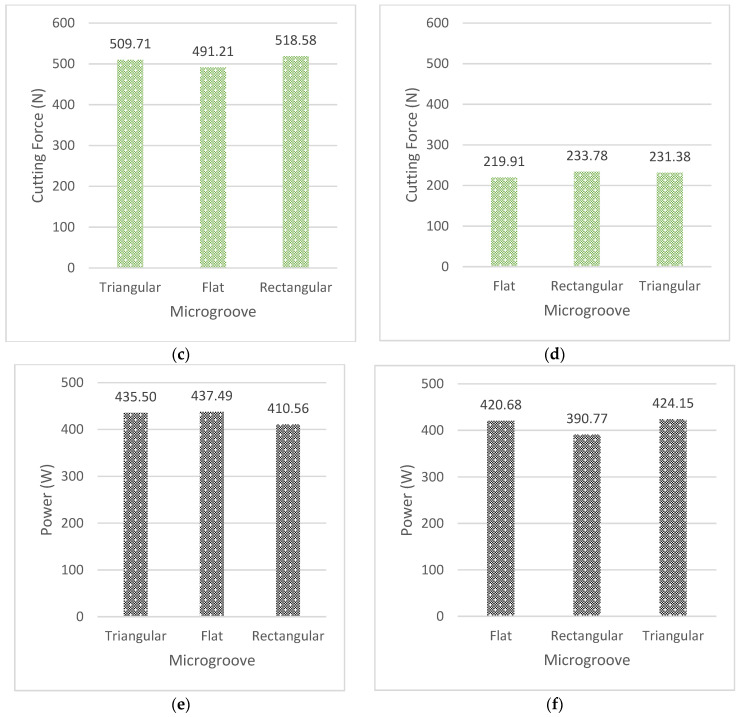
At cutting speed of 75 m/min: (**a**) cutting temperature at f = 0.3 mm/rev, (**b**) cutting temperature at f = 0.1 mm/rev, (**c**) cutting force (*Fx*) at f = 0.3 mm/rev, (**d**) cutting force (*Fx*) at f = 0.1 mm/rev, (**e**) cutting power at f = 0.3 mm/rev, (**f**) cutting power at f = 0.1 mm/rev.

To study the effect of the feed rate and micro-grooves on different cutting output responses, the cutting speed was kept constant. Firstly, the temperature is discussing here by altering the feed rate for the rectangular microgroove at a cutting speed of 75 m/min. A cutting temperature of 390 °C was recorded at 75 m/rev and 0.1 mm/rev feed rates, and 410.55 °C at 0.3 mm/rev feed rates. However, flat and triangular grooves at 75 m/min and 0.3 mm/rev showed a temperature of 437.493 °C and 435.5 °C, respectively. It is seen that more than 5% of the cutting temperature was dropped in the rectangular tools. However, when cutting forces and cutting power was observed the rectangular tool, this did not provide the optimum results. This is for the reason that a lower cutting temperature keeps the workpiece material hard by suppressing the thermal softening phenomenon. In addition to this, the comparison of cutting force showed that an approximately 55% decrease in force was reported at the lower feed rate of 0.1 mm/rev. Figure 12 shows the output responses at cutting speed of 75 m/min. Table 12 reported the values obtained at the constant cutting speed of 75 m/min with two feed levels of 0.1–0.3 mm/rev.

## 4. Conclusions 

The following conclusions were drawn from this simulation based only on numerical study. Due to the lack of resources, the scope of this study was kept to numerical analysis only. In future, more dedicated simulations will be performed to model the tool wear effects and experimental verification will also be conducted.

From the above discussion it is concluded that the use of rectangular microgroove decreases the temperature of the cutting tool by almost 10%, as compared with the flat cutting tool at a higher feed of 0.3 mm/rev or depth of cut excluding the 150 m/min condition, where it showed a decrease of 4%. The reason attributed to this is linked with the reduction in the coefficient of friction (COF).

The micro-grooves influenced the cutting temperatures on the rank of second after the cutting speed. The result of ANOVA showed that the micro-grooves affect the cutting temperature by 43.29%, and the cutting speed has the effect of 48.72%, which is a little higher than microgrooves.

From the signal to noise ratio and mean response plot, it was observed that the influence of -micro-grooves was at the second rank for the cutting forces and temperatures after the cutting speed. This means that the microgroove is a promising factor to influence forces and temperature.

The ANOVA technique is used to find the exact value of the percentage of the effecting factor on the total cutting power. The feed rate contributes more than 98% in power and microgroove affects that by 0.94%. 

The higher forces observed against the rectangular tool can be linked with the reason that the lower cutting temperature kept the workpiece material hard by suppressing the thermal softening phenomenon. However, when the overall machining performance was considered, rectangular micro-grooved tools provided comparatively better performance.

## Figures and Tables

**Table 1:** Parameters of Microgrooves.

Parameters	RT Tool	VT Tool
Width of groove (mm)	0.1	0.2
Depth of groove (mm)	0.05	0.05
Distance between groove (mm)	0.2	0.2
Radius at tip of cutting tool (mm)	0.015	0.015
Relief angle (degree)	10	10

**Table 2:** Taguchi design parameters for the cutting of work material.

Sr. No.	Cutting Speed (m/min)	Feed (mm/rev)	Microgroove
	V_c_	(t_o_)	
1	75	0.1	Flat
2	75	0.2	Rectangular
3	75	0.3	Triangular
4	150	0.1	Rectangular
5	150	0.2	Triangular
6	150	0.3	Flat
7	225	0.1	Triangular
8	225	0.2	Flat
9	225	0.3	Rectangular

**Table 3:** Cutting temperature: Response of signal to noise ratio.

Level	Cutting Speed m/min	Feed mm/rev	Micro Groove
1	−52.30	−52.89	−53.07
2	−53.10	−52.76	−52.33
3	−53.09	−52.85	−53.09
Delta	0.79	0.13	0.75
Rank	1	3	2

**Table 4:** Cutting temperature: mean response.

Level	Cutting Speed m/min	Feed mm/rev	Micro Groove
1	412.9	441.3	450.9
2	451.8	436.1	414.4
3	452.1	439.4	451.5
Delta	39.1	5.2	37.0
Rank	1	3	2

**Table 5:** Analysis of variance for temperature values.

Source	DF	Seq SS	Contribution	Adj SS	Adj MS	F-Value	*p*-Value
Cutting speed m/min	2	1.25	48.72%	1.25	0.62	7.03	0.12
Feed mm/rev	2	0.03	1.06%	0.03	0.01	0.15	0.87
Micro groove	2	1.11	43.29%	1.11	0.56	6.25	0.14
Error	2	0.18	6.93%	0.18	0.09		
Total	8	2.57	100.00%				

**Table 6:** Calculation of Chip Compression Ratio and Shear Angle.

Sr. No.	Cutting Speed (m/min)	Microgroove	Feed (mm/rev)	Undeformed Chip Thickness (mm)	Chip Ratio	Shear Angle (Degree)
	*Vc*		*to*	*tc*	*r*	∅
1	75	Flat	0.1	0.11	0.901	42.01
2	75	Rectangular	0.2	0.214	0.934	43.06
3	75	Triangular	0.3	0.325	0.923	42.70
4	150	Rectangular	0.1	0.123	0.813	39.11
5	150	Triangular	0.2	0.244	0.818	39.28
6	150	Flat	0.3	0.333	0.901	42.02
7	225	Triangular	0.1	0.129	0.775	37.78
8	225	Flat	0.2	0.221	0.903	42.08
9	225	Rectangular	0.3	0.326	0.920	42.62

**Table 7:** Cutting power, shear power, friction power and total power at different cutting conditions.

Sr. No.	Cutting Power W	Shear Velocity m/min	Shear Power W	Chip Velocity m/min	Friction Power W	Total Power W
	*Pc*	*Vs*	*Ps*	*Vf*	*Pf*	*Pt*
1	275.89	100.947	135.886	67.56	125.226	537.002
2	480.64	102.655	281.575	70.09	186.051	948.266
3	637.13	102.068	444.000	69.23	178.281	1259.411
4	557.2	193.319	277.950	121.95	227.032	1062.183
5	906.81	193.791	616.175	122.69	237.750	1760.736
6	1190.68	201.895	884.375	135.13	275.946	2351.001
7	813.24	284.687	446.737	174.42	284.099	1544.076
8	1277.58	303.149	884.137	203.16	355.259	2516.977
9	1996.16	305.773	1364.175	207.05	581.583	3941.919

**Table 8:** Total cutting power: Response of signal to noise (SN) ratio.

Level	Cutting Speed m/min	Feed mm/rev	Micro Groove
1	−50.90	−46.84	−50.35
2	−50.57	−51.18	−51.06
3	−50.63	−54.08	−50.69
Delta	0.33	7.23	0.71
Rank	3	1	2

**Table 9:** Total cutting power: Mean response.

Level	Cutting Speed m/min	Feed mm/rev	Micro Groove
1	371.4	219.9	345.6
2	354.0	362.6	379.9
3	363.3	506.1	363.1
Delta	17.4	286.2	34.3
Rank	3	1	2

**Table 10:** Analysis of variance for total cutting power value.

Source	DF	Seq SS	Contribution	Adj SS	Adj MS	F-Value	*p*-Value
Cutting speed m/min	2	0.181	0.22%	0.181	0.090	1.57	0.389
Feed mm/rev	2	79.450	98.69%	79.495	39.747	691.12	0.001
Micro groove	2	0.760	0.94%	0.760	0.378	6.60	0.132
Error	2	0.115	0.14%	0.115	0.057		
Total	8	80.550	100.00%				

**Table 11:** Analysis of microgroove at different cutting speed and constant feed rate.

Microgroove	Cutting Speed (m/min)	Feed Rate (mm/rev)	*Fx* (N)	*Fy* (N)	Power (W)	Temperature (°C)
Rectangular	225	0.3	523.31	168.53	1996.16	421.55
Flat	225	0.3	455.46	109.39	1707.98	470.55
Triangle	225	0.3	489.43	119.34	1835.36	470.66
Triangular	75	0.3	509.71	154.51	637.13	435.5
Flat	75	0.3	491.208	151.298	614.010	437.493
Rectangular	75	0.3	518.582	170.725	648.227	410.555

**Table 12:** Analysis of cutting parameters at different feed rate but constant cutting speed.

Microgroove	Cutting Speed m/min	Feed Rate mm/rev	*Fx* (N)	*Fy* (N)	Power (W)	Temperature (°C)
Flat	75	0.1	219.91	111.201	275.89	420.68
Rectangular	75	0.1	233.77	124.373	292.224	390.771
Triangle	75	0.1	231.38	120.37	289.231	424.148
Triangular	75	0.3	509.71	154.51	637.13	435.5
Flat	75	0.3	491.208	151.298	614.011	437.493
Rectangular	75	0.3	518.582	170.725	648.227	410.555

## Data Availability

Can be requested to the author at sxpcad@rit.edu.
